# P-791. Contribution of tuberculin skin test for diagnosing tuberculosis disease in pediatric patients in Bangladesh

**DOI:** 10.1093/ofid/ofae631.983

**Published:** 2025-01-29

**Authors:** Giordano Sosa Soto, Farzana Hossain, Toufiq Rahman, Mahfuzur Rahman, Suchitra Kulkarni, Tapash Roy, Amyn Malik, Meredith Brooks

**Affiliations:** Boston Children's Hospital/Harvard Medical School, Boston, Massachusetts; IRD Bangladesh, Dahka, Dhaka, Bangladesh; Stop TB Partnership, Geneva, Geneve, Switzerland; IRD Bangladesh, Dahka, Dhaka, Bangladesh; Boston Medical Center, Boston, Massachusetts; IRD Global, Bangladesh, Dhaka, Dhaka, Bangladesh; UT Southwestern Medical Center, Dallas, Texas; Boston University School of Public Health, Boston, Massachusetts

## Abstract

**Background:**

Bangladesh has a high tuberculosis (TB) burden, accounting for 4% of pediatric patients estimated to have TB globally. Obtaining microbiological confirmation of disease in children is challenging; in lieu of this, diagnosis often relies on the presence of symptoms, exposure history, physical examination, and a variety of other test results, such as chest x-rays and tuberculin skin tests (TST). We evaluate the contribution of TST to pediatric TB diagnoses.

**Figure:**
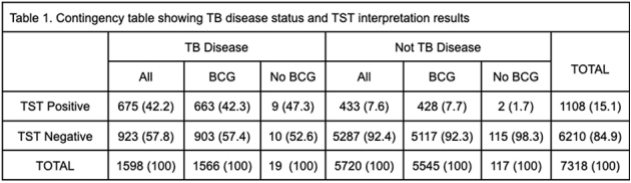

**Methods:**

An enhanced patient-finding initiative was implemented across 109 facilities in Mymensingh division, Bangladesh (2018-2021) to improve pediatric TB detection. Among children who screened positive on systematic verbal symptom screening and whose subsequent evaluation for TB disease included TST, we report the discriminatory properties of TST for TB diagnosis. We compare patient characteristics between children with positive and negative TST results using chi-squared/Fisher’s exact test.

**Figure:**
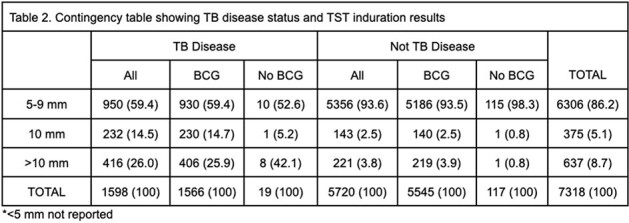

**Results:**

Of 20,209 children who screened positive on verbal symptom assessment and were further evaluated for disease, 7,318 (36.2%) patients had TST, of which 1598 (21.8%) were diagnosed with TB disease. Discriminatory properties of TST were: sensitivity of 42.2% (95% Confidence Interval: 39.8-44.7%), specificity of 92.4% (91.7-93.1%), PPV of 60.9% (58.3-63.4%), and NPV of 85.1% (84.6-85.7%) (Table 1). A total of 648 (40.6%) children with TB and 364 (6.4%) children without TB had an induration diameter of >10mm (Table 2). Among children diagnosed with TB, fewer males (p=0.01) and children aged 5-9 years (p< 0.01), and more children aged 10-14 years (p< 0.01), underweight (p< 0.01), presenting with night sweats (p=0.02) and decreased appetite (p=0.04), and had a chest x-ray (p< 0.01) or smear (p=0.01) suggestive of TB disease had a positive-TST result compared to children with negative-TST results. Among children with a negative TST result, those who were underweight were diagnosed with TB more often than those not underweight (p< 0.01) (Table 3).

**Figure:**
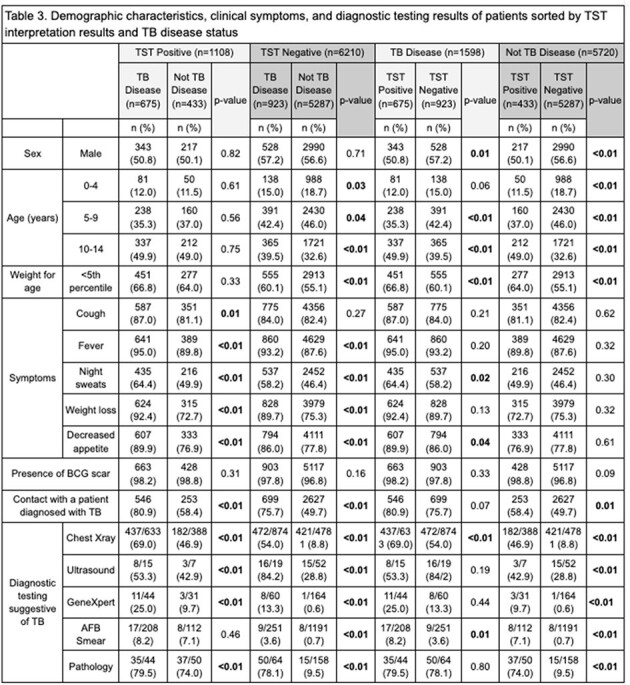

**Conclusion:**

TST can be a clinically helpful part of diagnostic algorithms for children. Given its high specificity and NPV, TST can be combined with an evaluation of clinical symptoms and diagnostic studies to help diagnose or rule out disease in children at risk of TB.

**Disclosures:**

**Amyn Malik, MBBS, MPH, PhD**, Analysis Group, Inc: Former Employee. Worked as a consultant with Pharma and Biotech for research studies

